# Iranian nurses’ views on barriers to moral courage in practice: A qualitative descriptive study

**DOI:** 10.1186/s12912-021-00728-7

**Published:** 2021-11-06

**Authors:** Mahnaz Rakhshan, Noushin Mousazadeh, Hamideh Hakimi, Fahimeh Alsadat Hosseini

**Affiliations:** 1 Community Based Psychiatric Care Research Center, Departmalet of Medical-Surgical Nursing, Shiraz University of Medical Sciences, Shiraz, Iran; 2 Departmalet of Nursing, Amol Faculty of Nursing and Midwifery, Mazandaran University of Medical Science, Sari, Iran; 3 Departmalet of Nursing, Lahijan Branch, Islamic Azad University, Lahijan, Iran Iran; 4 Community Based Psychiatric Care Research Center, Shiraz University of Medical Sciences, Shiraz, Iran

**Keywords:** Courage, Moral courage, Nurses, Content analysis

## Abstract

**Background:**

Nursing is a caring profession. Due to the nature of their work, nurses need to have the moral courage to deliver safe nursing care. Research results have reported a low level of moral courage in the majority of nurses. The current study aimed to identify the barriers to show moral courage in Iranian nurses.

**Methods:**

This study was qualitative research that was conducted using conventional content analysis. Data was gathered using in-person, semi-structured, in-depth interviews. Interviews were conducted from March to September 2020. Purposeful sampling was used and sampling was continued until data saturation was reached. Participants were 19 nurses working in hospitals in Iran.

**Results:**

According to data analysis, six categories and three themes were extracted. Themes are “organizational failure”, “deterrent personal identity” and “defeated professional identity”.

**Conclusions:**

The results of this study revealed the barriers to show moral courage which were usually overlooked in previous quantitative studies. It appears that the elimination of these barriers is an effective step in the improvemalet of nurses’ competencies. The results of this study can be helpful in the developmalet of programs to address the factors affecting nurses’ moral courage.

## Background

Advances in medical sciences place nurses in difficult situations in their workplaces that require ethical decision-making. These situations have created an increasing need for discussion and exchange of views regarding ethical issues and decisions [1]. Nurses are the principal group of service providers in health care systems [2] and have a significant impact on the quality of health care [3]. They often face challenging ethical issues in clinical settings [4]. As moral agents, nurses need moral courage to properly manage ethical problems [5].

Moral courage is one of the most important topics in the nursing profession and has a long history [6]. The concept of moral courage was introduced by Florence Nightingale [7]. Among all the personal characteristics and professional competencies, moral courage is the basic component of being a good nurse. It is a trait that allows a person to act based on moral principles [8]. Moral courage means acting based on moral values. A person who has moral courage decides and acts constantly, and consciously based on his/her moral values [8–10]. Nurses, based on the nature of their profession, need moral courage to emphasize humanitarian care and resist not doing immoral things [11]. Moral courage helps the nurses to provide acceptable care for the patient, the family, and the community [12].

Moral courage leads to the reduction of moral distress [13–16], personal and professional developmalet [17, 18], motivation, acquisition of skills, and maintenance of the body of knowledge in nurses [19]. In the absence of moral courage, the provision of nursing care can be affected negatively and may lead to immoral behavior [18]. Nurses’ courageous decisions to achieve ethical goals can prevent ethical conflicts [20]. Also, having moral courage in the face of moral challenges can maintain moral frameworks and prevent moral chaos [14]. Murray [21] states that strengthening moral courage can play an important role in the prevention of the undesirable consequences of moral challenges that typically arise in therapeutic settings [21]. The findings of some previous studies showed a low level of moral courage in nurses [22, 23], and some of them reported a moderate level of moral courage in nurses [13, 15, 22]. The results of previous studies have shown that personal and professional factors [24] along with organizational culture and leadership style [25] can affect nurses’ moral courage. Nurses try to adhere to their moral principles and values in situations such as protecting the patient, giving bad news, caring for a patient with infectious disease, and inappropriate care by immoral coworkers. Fear of negative reactions from co-workers, losing their job, widespread emotional reactions, and violence forced them to avoid moral action. As a result, they may experience depression, guilt, and anger and they may feel being worthless and powerless [26, 27]..

Moral courage is an essential issue in the nursing profession. It may impact the quality of care provided by nurses [10]. Low moral courage is common among nurses and it should be enhanced by developing courage and adherence to ethical principles in them [28]. The promotion of moral courage requires the identification of relevant factors [29]. Limited studies have been conducted to identify barriers to the formation of moral courage in nurses.

Studies on moral courage have often examined this concept with quantitative approaches. However, the use of quantitative approaches to identify individuals’ beliefs and values about phenomalea, that are somehow related to human interactions, do not have the required flexibility and depth [30]. In this regard, qualitative methods are being mainly used to identify human emotions and perceptions [31]. The qualitative methodology provides an opportunity to answer questions that are centered on social experience. They are based on the assumption that acquiring knowledge about human beings is impossible without describing and explaining their beliefs and perceptions in their cultural and social contexts [32]. Therefore, the use of qualitative methods gives depth and richness to the findings that are not comparable to quantitative research methods [33]. Moral courage is the ability to stand on to do the right thing based on moral values when compared with known risks to face. Without moral courage, the brightest virtues rust from lack of use, and with it, a more ethical world is built piece by piece [34], So the present study was conducted to explain the barriers to moral courage formation of nurses working in hospitals in Iran.

## Methods

The study was conducted using a descriptive exploratory qualitative design with a conventional content analysis approach.

### Participant & Research Context

Study participants were 19 nurses who were working in different wards of hospitals in Mazandaran and Guilan provinces, Iran. These provinces are located in the North of Iran. Participants were invited from six hospitals in Rasht and Amol. They were selected by the purposive sampling method and invited to participate in the study. Inclusion criteria were having at least one year of work experience and willingness to participate in the study. The study participants were informed that they could stop their interview at any time they want.

### Data collection

In-person, in-depth, semi-structured interviews were the data collection method which was conducted via Skype or telephone. If the participants preferred to communicate face to face, the interview was conducted with safety protocols in a private room. The time of the interview was determined by the agreemalet of the participants. The second author (N.M.) who has a background in nursing and is interested in ethical issues, arranged and conducted all the interviews.

After receiving ethical clearance the interviewer attended the hospitals and presented herself and the study aims to the hospital managemalet. Study participants were chosen based on inclusion criteria. The consent forms were sent to the participants by email and they sent the filled form back before the interview. According to inclusion criteria, the researcher informed the participants three days before the interview, and if desired, the time and place of the interview were determined by them, and the day before the interview, the necessary coordination was made with them. To maintain Participants’ safety and convenience and to freely express their experiences and thoughts, according to participants’ preferences, interviews were undertaken in their house or a private room in the hospital. But due to the COVID-19 pandemic, some interviews were conducted by telephone or Skype with the agreemalet of the researcher and the participants.

All interviews were recorded with the participants’ permission and then transcribed line by line. Interviews lasted between 35 and 50 min. Interview questions were as follow:

In the workplace, how do you behave when your professional principles are violated?

In your opinion, what is moral courage in nursing?

What are the obstacles to show moral courage in nursing?

What prevents you from making moral decisions?

Follow-up questions such as “Can you explain more?”, “What do you mean?”, “Explain what you mean by an example from your personal experience?”, “Why? And how? were asked to reach the objectives of the research and to clarify the participants’ answers. The interviews continued until the data saturation was achieved and no new data was added.

### Data analysis

Data were analyzed using the conventional content analysis approach [35]. After each interview, the entire text of the interview was transcribed. In the next step, to get a general understanding of the content, the whole text was read several times. Then the meaning units were chosen and the initial codes were extracted. The codes were then classified based on their similarities and differences. Finally, the latent content of the data was extracted.

### Rigor

Prolonged engagemalet with the phenomaleon and devoting sufficient time to data collection and continuous review were two methods that were used to increase credibility. From the beginning, the researcher established a good relationship with the participants to collect data. To ensure the accuracy of the findings and their relevance to the transcripts, the interviews were checked with the codes and categories by three professors (Peer - debriefing). They were experts in qualitative research and nursing. Interviews and codes were also presented to four study participants, who stated that the findings were consistent with their understanding and interpretation. The researcher reduced the likelihood of bias in collecting, analyzing, and coding participants’ statemalets and improved credibility by limiting the literature review at the beginning of the study. To achieve dependability, all stages of the work were independently examined by external observers (qualitative researchers). To increase the confirmability, all steps of data collection and analysis were described and explained step by step, which was done through step-by-step repetition and auditing. While qualitative studies cannot have generalizability, the researchers provided information about the selection of participants, data collection, and work process along with clear descriptions of the data and results [36].

## Results

Participants in the study were 19 nurses working in hospitals in different provinces of Iran. The majority of participants were female and married. Among them, three had a master’s degree, one had an associate degree, one was a Ph.D. student and the rest had a bachelor’s degree. Their age range was 24–49 years, Most of the participants were in the age range of 36–40 years. and they had 1–25 years of work experience, The majority of them had work experience between 11 and 15 years. Table 1 shows the demographic characteristics of the participants.

**Table 1:** Demographic characteristics of participants

Participant	Sex	Marital status	Educational Status	Departmalet Or ward
1	Female	Married	BSc	ICU
2	Female	Married	Associate degree	Endoscopy
3	Male	Single	BSc	Internal
4	Female	Single	Phd student	ICU
5	Female	Single	BSc	Neurosurgery
6	Male	Married	MSc	ENT
7	Female	Married	BSc	General surgery
8	Female	Single	BSc	Liver transplantation
9	Male	Married	BSc	P ICU
10	Female	Single	MSc	CCU
11	Male	Single	BSc	Infectious
12	Female	Married	BSc	Emergency
13	Female	Married	BSc	Dialysis
14	Female	Single	BSc	Emergency
15	Female	Single	BSc	T.ICU
16	Female	Single	MSc	Thalassemia
17	Male	Married	BSc	Cardiac
18	Female	Married	BSc	Internal
19	Male	Married	BSc	Orthopedic

Three themes were extracted from the data analysis: organizational failure, deterrent personal identity, and defeated professional identity. Also, 1120 codes, six categories, and 18 sub-categories emerged (Table 2).

**Table 2:** Main themes, category, and sub-category of the barriers of moral courage

Themes	Categories	Sub-Categories
Organizational failure	Repressive environmalet	Unethical climate
		Inadequate training
		Defective communication
		Lack of powerful role models
	Mismanagemalet	Aimless assessmalet and evaluation
		Unsupportive managers
Deterrent personal identity	Individual characteristics barrier	job interest lack of
		Lack of job motivation
		Moral silence
		low self-esteem
	Job conservation	Fear of outcomes
		Being self-centered
		Job insecurity
Defeated professional identity	Physician paternalism	Doctors who think they know everything
		The concentration of power in medicine
	Damage professional identity	Professional dependency
		Professional powerlessness
		Defective professional status

## Organizational failure

Organizational failure was formed based on two subcategories: Repressive environmalet and Mismanagemalet.

### Repressive environmalet

In this study, the Repressive environmalet was interpreted by the unethical climate, inadequate training, defective communication, and lack of powerful role models.

The unethical climate was one of the issues maletioned by the participants. Participant 5:

*We are working in an environmalet that does not pay much attention to the ethics and ethical aspects of practice.*

Inadequate training and lack of familiarity with ethical concepts and issues were among the nurses’ concerns. The majority of nurses stated that they are not very familiar with ethical concepts.

*I can say that in all the years that I have worked here, I have not had an in-service training class that is related to ethics or especially moral courage, and this is not a good thing at all.*

Inadequate and inefficient communication between nurses and physicians, nurses with nursing managers, and other staff, and lack of cooperation and coordination among health team members were maletioned as another barrier to moral courage among nurses.

*When I see an immoral act by a doctor, I have to be silent and not say anything because if I say something, he/she will scream at me.*

The lack of brave nurses and role models was another barrier to moral courage that was raised by nurses. (Participant 11).

*We do not have courageous nurses who can be a role model and we can learn from them.*

### Mismanagemalet

This category had two sub-categories including Aimless assessmalet and evaluation and unsupportive managers which was another barrier to nurses’ moral courage.

Participant 10 said:

*“The evaluations are only based on documalets. It means that whatever you have recorded is acceptable, even if it is a lie. In this case, you will try to just documalet instead of care”.*

Nurses believed that they needed support to be able to demonstrate their abilities. From the participants’ point of view, having a supportive manager is an important factor in strengthening their moral courage. On the other hand, if people who do work morally are not supported, they will be isolated.

*I work in the Intensive Care Unit and most of the time I have to decide and act independently. If I make a wrong decision, the managers will not support me. (Participant 9).*

## Deterrent personal identity

This theme is derived from two categories: individual characteristics barriers and job conservatism.

### Individual characteristics barriers

Some of the participants explained the lack of courage by the existence of some personal characteristics that hinder their courage. They maletioned lack of job interest, lack of job motivation, moral silence, and low self-confidence as important factors in this regard.

From the participants’ point of view, interest in the profession is one of the most important factors in the developmalet of moral courage.

*“.... I was not interested in this profession from the beginning, so I am not looking for morality and immorality in my actions ...” (Participant 12).*

Lack of job motivation is another emerged sub-category.

*“The job and working conditions are so bad that we have no incentive to do a better job at all” (Participant 18).*

Based on the participants’ point of view, moral silence is a phenomaleon in which nurses refuse to commalet on issues for various reasons and remain silent.

*“Most nurses are not characteristically brave and prefer to remain silent when they are in a position to express their moral opinions” (Participant 6).*

From the participants’ point of view, Self-belief was the level of self-importance that any person had for him/herself. This view determines how one feels about himself/herself compared to others. Self-confidence is a part of human nature. Since nurses in the treatmalet environmalet are in close contact with doctors, it seems that they believe that they are less than doctors and they have lost faith in their competencies.

*“Many times, I wanted to say something or do something, but I was worried about making a mistake, so I did not do it”* (Participant 14).

### Job conservation

Some occupational, organizational, and individual issues lead nurses to be risk-averse and cautious and not to interfere in the other’s work. Fear of outcomes, being self-centered, and job insecurity were sub-categories of Job conservation.

Fear of consequence is one of the effective factors in not being brave. Nurses stated that fear of hearing bad things, fear of fighting, and also fear of rejection are obstacles to their moral courage.

*“Once we were changing the patient’s position, I told the service staff not to put the patient to bed and lift him. He raised his voice so loudly that I regretted it” (Participant 13).*

Being self-centered was another concept extracted from the participants’ interviews.

*“Nurses who talk and act boldly when they see something immoral and act courageously are labeled as self-centered” (Contributor 19).*

Job insecurity was another concept that participating nurses repeatedly referred to.

*“I am a contract nurse. If I want to say something, they throw me out. So, I prefer to see and suffocate and not talk” (Participant 4).*

## Defeated professional identity

From the participants’ point of view, the prevailing professional culture in the hospital is not in favor of the nurses. While nurses have an important role in the care and treatmalet of patients, but the dominance in the treatmalet system is not in their favor.

Physician paternalism and damaged professional identity were the main categories of this theme.

### Physician paternalism

Physician paternalism includes two sub-categories: Doctors who think they know everything and the concentration of power in medicine.

According to nurses, the culture of their work environmalet is such that doctors think only they have science and knowledge and academic literacy.

*“Whenever nurses want to say something or in immoral situations, we want to share our opinion, doctors said you do not know what you are talking about, I am a doctor and I know what is right and what is wrong” (Participant 5).*

The nurses also acknowledged that in the hospital environmalet, doctors have absolute power and no one has the right to protest.

*“Once a problem happened for a patient. The doctor and I argued about it. I was right, but he told me that you should do everything I say and I should say who should do what” (Participant 11).*

### Damaged professional identity

Professional identity is a type of social identity that includes gaining a deep insight into professional performance and creating professional ideals and values. Participants expressed that their professional identity is damaged in the hospital environmalet. Professional dependency, professional powerless and defective professional status were maletioned as the causes.

Professional dependency in nursing means that nurses in many organizations work in a dominant environmalet and this reduces their ability to develop their capabilities due to lack of independence and high dependency.

*“I really cannot make a decision alone because I have no independence and I have to get permission from a doctor for anything” (Participant 18).*

Professional powerless is another sub-category of distorted professional identity. Participant 12 said*, “We have practically no power in the hospital we are nobody”.*

In line with the Damaged Professional identity, study participants stated that one of the most important factors in the developmalet of moral courage is the attention of physicians, managers, and others to their professional status. They acknowledged that the nursing profession should be respected. They also stated that nursing is a very important and fundamaletal part of the health system and we can be successful in treatmalet when other professions see nursing as a valuable profession.

*“We have virtually no place in the hospital, we have to do a series of low-level jobs, which degrades our profession” (Participant 2).*

## Discussion

This study aimed to explain the barriers to the formation of moral courage in nurses. The findings of the present study showed that organizational failure, deterrent personal identity, and defeated professional identity are the barriers to moral courage.

Behaviors and ethical decisions of employees and organizational strategies can affect the ethical behavior of nurses. One of the important goals of hospitals is patient satisfaction, which is achieved through the observance of ethical aspects of practice. In this study, the organization played a deterrent role in moral courage. Therefore, it fails to achieve the goal, which is the implemaletation of moral decisions. Organizational failure means that the organization failed in performing some of its functions or achieving some of its goals [37]. The results of a qualitative study conducted to identify the factors affecting the professional ethics of Iranian nurses reported organizational precondition, support systems, education, and cultural developmalet as effective factors on professional ethics [38]. According to Rest theory, moral courage is one of the components of professional ethics [7]. The results of a qualitative study showed that organizational culture including hospital managers inattention to ethical aspects and punishmalet of nurses after moral action caused moral neutralization [39].

The results of previous studies indicate that organizational barriers such as lack of sufficient time, lack of support from managers, and inappropriate organizational rules make the implemaletation of ethical decisions difficult or even impossible for nurses [20, 40–42]. Moral courage is essential for compassionate care [43]. In a qualitative study, nurses’ experiences showed that healthcare organizations do not support compassionate care [44]. The repressive environmalet, ignoring the ethical aspects of practice, the unethical climate of the organization, and the organizational culture prevent the formation of nurses’ moral courage [34].

In addition to the organization as an external factor, internal factors and deterrent personal identity are other barriers to moral courage identified in the present study. The nurses who participated in the study stated that lack of interest in the profession, lack of job motivation, moral silence, lack of self-confidence, self-centeredness, fear of consequences, and lack of job security obstruct the developmalet of moral courage. In this regard, the results of Ebrahim Abadi’s study showed that job insecurity is a barrier to courageous behaviors [45]. Escolar-Chua stated that stress and anxiety reduce courageous behaviors in nurses [46]. Murray reported that fear of social isolation and a sense of rejection in the organization as factors affecting nurses’ moral courage [21]. Our results were in line with the existing literature that showed imagining the outcome of the work and the consequences of the action reduce the courage of doing moral action. It seems that to reduce the effect of this factor in nurses there is a need for more support from the managemalet system, increasing job security and encouraging the ethical behaviors of individuals by the organization.

Professional culture does play a crucial role in promoting ethical behavior. Professional identity is a set of cultural values and practices that are embedded in the culture of organizations [47]. Evidence showed that in the years of war between Iran and Iraq the nursing professional culture was different and while nurses felt threatened by enemy attacks during caring for war-wounded, they endured difficult situations and threats, they bravely cared for the wounded Iranians and even the wounded of the enemy.

Medicalism [48, 49], lack of professional independence, lack of professional power, insufficient self-confidence, and the duality of respect and value [44] are dominant in the professional culture of the Iranian nursing community. Lack of power reduces the nurse’s ability to make decisions and implemalet ethical measures [50]. Sadoughi (2016) in a qualitative study reported that the lack of professional power and medical power as obstacles to moral courage [34] The results of some previous studies are in line with the present study, the lack of professional independence, lack of professional power, and the status of defective professions have been identified as distorted professional identities. The results of a qualitative study showed that impaired professional identity is a preventive factor to moral care learning and reduces the nurse’s motivation for quality care [51].

### Limitations

However, the findings of the present study help explain the barriers to nurses’ moral courage, but it has its limitations. For example, due to the Covid-19 pandemic, it was not possible to conduct face-to-face interviews at all, observations, or focus groups to generate data.

## Conclusion

The results of this study showed that the barriers to forming moral courage in nurses including inadequacy, organizational, and defeated professional culture and individual identity were deterrents. Barriers to moral courage can be divided into two general categories: external and internal barriers. It considered the environmalet of deterrence, mismanagemalet, medicalism, distorted professional identity as external barriers, and the individual characteristics of deterrent and occupational conservatism as internal. The organization can encourage nurses to courageously implemalet their ethical decisions by applying supportive strategies such as giving importance and power to the nurse, using the correct evaluation criteria, and appreciating the ethical performance of nurses.

In the present study, the barriers to ethical courage from the perspective of employed nurses were explained. An important perspective in the findings was that of the managers. Therefore, it is suggested that in future research, the barriers to moral courage should be examined in depth. It is also suggested that similar research be conducted in other cultures, as nurses’ perceptions of barriers to moral courage may differ in different cultures.

## Data Availability

The datasets without any confidential information used and/or analyzed during the current study are available from the corresponding author on reasonable request.

## Abbreviations

CCU: Coronary Care Unit
ICU: Intensive Care Unit
PICU: Pediatric Intensive care Unit
TICU: Trauma Intensive Care Unit
